# Does the effect of gender modify the relationship between deprivation and mortality?

**DOI:** 10.1186/1471-2458-12-574

**Published:** 2012-07-30

**Authors:** Natalia Salcedo, Marc Saez, Basili Bragulat, Carme Saurina

**Affiliations:** 1 Research Group on Statistics, Econometrics and Health (GRECS), University of Girona, Girona, Spain; 2 Research Group on Statistics, Econometrics and Health (GRECS), CIBER of Epidemiology and Public Health (CIBERESP), University of Girona, Campus de Montilivi, 17071, Girona, Spain

**Keywords:** Deprivation indexes, Distance indicator DP_2_, Ecological regression, Standardized mortality ratio, Hierarchical Bayesian models, Robust

## Abstract

**Background:**

In this study we propose improvements to the method of elaborating deprivation indexes. First, in the selection of the variables, we incorporated a wider range of both objective and subjective measures. Second, in the statistical methodology, we used a distance indicator instead of the standard aggregating method principal component analysis. Third, we propose another methodological improvement, which consists in the use of a more robust statistical method to assess the relationship between deprivation and health responses in ecological regressions.

**Methods:**

We conducted an ecological small-area analysis based on the residents of the Metropolitan region of Barcelona in the period 1994–2007. Standardized mortality rates, stratified by sex, were studied for four mortality causes: tumor of the bronquial, lung and trachea, diabetes mellitus type II, breast cancer, and prostate cancer. Socioeconomic conditions were summarized using a deprivation index. Sixteen socio-demographic variables available in the Spanish Census of Population and Housing were included. The deprivation index was constructed by aggregating the above-mentioned variables using the distance indicator, DP_2_. For the estimation of the ecological regression we used hierarchical Bayesian models with some improvements.

**Results:**

At greater deprivation, there is an increased risk of dying from diabetes for both sexes and of dying from lung cancer for men. On the other hand, at greater deprivation, there is a decreased risk of dying from breast cancer and lung cancer for women. We did not find a clear relationship in the case of prostate cancer (presenting an increased risk but only in the second quintile of deprivation).

**Conclusions:**

We believe our results were obtained using a more robust methodology. First off, we have built a better index that allows us to directly collect the variability of contextual variables without having to use arbitrary weights. Secondly, we have solved two major problems that are present in spatial ecological regressions, i.e. those that use spatial data and, consequently, perform a spatial adjustment in order to obtain consistent estimators.

## Background

Deprivation has been defined by Townsend as ‘a state of observable and demonstrable disadvantage relative to the local community or the wider society or nation to which an individual, family or group belong to’ [1]. It is a concept that has two distinguishable domains, material and social circumstances. The former relates to diet, health, clothing, housing, household facilities, environment and work. The latter is more difficult to measure as it relates to different forms of relationships, such as community integration, leisure, and formal participation in social institutions [1].

A deprivation index (DI) is recognized as a composite measure, where no single variable can be said to measure it but rather a number of variables contribute in some way [2]. Area-level indicators are commonly used to evaluate the geographical distribution of socioeconomic inequalities in health [3-6]. Area-level indicators have been used as proxies for individual-level data when individual measures are not available (i.e. data from census is only provided at aggregated levels for reasons of confidentiality) [7].

Approaches to ecological small-area analysis propose using the smallest practicable spatial scale for any study on inequalities in health [8,9]. This diminishes the ecological bias, as the analysis is closer to the individual level [8,10,11]. Most studies that have focused on area-level social factors use geographical boundaries, developed for censuses, as proxies for actual communities or neighborhoods [6]. Administrative boundaries allow for routinely collected data to be available at the smallest scale. In Spain, census tracts, the smallest administrative unit, tend to have a mean population of 1,000 subjects [12].

There are three main methods for constructing Deprivation Indices (DIs) [13,14]. First, standardized z-scores or log transformations of a priori selected variables. This was a very popular method up until the late 1980´s [15,16]. The second method is the use of Principal Component Analysis (PCA) which has been the principal method used over the last twenty years. PCA transforms a number of possibly correlated variables into uncorrelated variables and permits the extraction of a smaller number of uncorrelated variables called principal components that collect a large percentage of the total variance contained in the original data which are then used to measure socio-economic status [17-20]. The third method (the least common) uses feedback from health experts to assign weights to the selected variables [21-24].

The increased use of DIs has allowed for the identification of deprived areas and their association to morbidity and mortality, living in deprived areas is an effect modifier. In fact, some studies confirm that people living in deprived areas have higher rates of ill health and mortality [4,5,8,25,26]. However, material deprivation is manifested in higher rates of mortality differently by gender [27,28]. This might be due in part to the fact that men and women perceive their environment differently [29-31], they may be exposed to different local environments [32], and their vulnerability to aspects in the local environment may vary [33].

In this study we propose improvements to the method of elaborating DIs. First, in the selection of the variables, we incorporated a wider range of both objective and subjective measures which makes for a more complete DI. Several studies that analyzed deprivation-associated excess mortality found that men had higher rates of excess mortality than women [27,28]. A large proportion of excess deaths were found in diet-related causes of death in females and smoking and alcohol consumption in males [27]. However, inferences about gender differences in relation to deprivation and mortality are sensitive to the choice of inequality measures used [34-36]. Making it important to incorporate indicators that are gender sensitive (i.e. residential/ environmental indicators as they are strongly related to women’s health and individual economic activity indicators as they are associated to men’s health) [32]. The strength of association between socio-economic and health measures tend to be greater for men than for women [35]. Thus, contextual indicators are crucial if we want to have a comparative measure between men and women’s health.

Studies have shown that neighborhood context influences self-rated health, especially in women, beyond the effects of individual factors [33,37]. Neighborhoods with worse socioeconomic conditions have a negative effect on health [38]. Additionally residential environment may be more important for women’s health while individual economic activity is more important for men’s health [32]. Other studies have found that occupational factors are more important for men’s health whereas the home environment is more important for women’s health [39].

Second, in the statistical methodology, we used a distance indicator (Pena Distance or DP_2_) instead of the leading aggregating method PCA. DP_2_ overcomes several limitations of PCA, for instance, aggregating variables expressed in different units of measurement, arbitrary weights, and duplicate information [40-45]. The greatest advantage of DP_2_ with respect to PCA consists on how each handles redundant information. Thus, while in PCA the first component, i.e. the DI, is composed of those variables that capture greater variability it leaves some variables out of the index. In DP_2_ the index is composed of all the variables, leaving out of the index only the redundant information but including each and every one of the variables. Thus, in principle, DP_2_ would collect more variability [45].

In addition, we propose another methodological improvement, which consists in the use of a more robust statistical method to assess the relationship between deprivation and health responses in ecological regressions.

In this sense, we focused on mortality from trachea, bronchial and lung cancer and diabetes, because there is certain evidence that gender could act as an effect modifier in the relationship with deprivation, and breast and prostate cancer, given their known association with deprivation. Several studies have shown a positive association between lung cancer mortality and deprivation for men and a negative association for women [18,28,46]. This is explained because in men, lower socioeconomic status implies a higher prevalence of smoking, which leads to an increased risk of lung cancer mortality in individuals with lower socioeconomic status. On the other hand, in women, those with higher socioeconomic status are those with a higher prevalence of smoking, leading to an increased risk of dying from that cause in individuals with higher socioeconomic status. Regarding diabetes, those individuals with high educational levels have a significantly lower risk of dying from this cause (odds ratio, OR = 0.66) compared to those with low educational levels [47]. In the Whitehall study [48], we observed a similar gradient for mortality from diabetes mellitus than with the other pathologies studied; higher mortality from diabetes mellitus in individuals who were in the lowest levels of the hierarchy. This excess mortality appeared to be due to the increased frequency of cardiovascular risk in this type of study group. In addition, variations in mortality have also been observed for diabetes mellitus type II (DM2) at the residence area level. Thus, individuals living in more deprived areas tend to have worse lifestyle (higher values of body mass index –BMI-, greater prevalence of smoking) which could lead to worse glycemic control and greater number of complications associated with diabetes mellitus. As in the case of lung cancer mortality, this association could be modified by sex.

However, the results from the Spanish literature, at least in those that use a spatial adjustment [18,28,46] do not systematically coincide with each other; particularly for prostate cancer and breast cancer. Diabetes mortality has shown a positive association with deprivation for both men [28,49] and women [49]. Borrell *et al.*[18] and Puigpinós-Riera *et al.*[46] found a positive association for breast cancer in Alicante (relative risk, RR = 1.55 95% confidence interval, 95% CI:1.04-2.19) yet the majority of other cities showed a negative association, not statistically significant, except for Vigo (RR = 0.54 95% CI 0.33-0.84). Benach *et al.*[27] found a negative association for breast cancer and deprivation with Index 1, which measured deprivation through unemployment, illiteracy and low social class; however, found no association with Index 2, measured deprivation through overcrowding and a small component of unemployment and illiteracy. For prostate cancer, some studies found no clear association between mortality risk and deprivation [46]. Benach *et al.*[27] found a negative association for prostate cancer and deprivation with Index 1; however, found no association with Index 2.

Our hypothesis is that the differences found in the literature are caused mainly by the lack of robustness of the statistical methodology applied and the lack of robustness of the spatial adjustment applied. Therefore, our objective in this paper is to evaluate a more robust methodology, in the way of developing DIs, the selection of component variables and how to build them, and, above all, in the statistical method applied to analyze how the effect of gender modifies the relationship between deprivation and mortality.

## Methods

### Design and study population

Most countries in the world periodically carry out population censuses that gather information on socio-demographic characteristics of the population resident in a country. In Spain, the Spanish Census of Population and Housing (CPH) conducted in 2001 reported nearly 41 million inhabitants. The province capital of Barcelona had over 1.5 million inhabitants and an average number of 1,201.33 inhabitants per census tract (standard deviation, SD, 526.66; Median 1,085.50). For this study, we conducted an ecological small-area analysis based on the residents of the Metropolitan region of Barcelona. A total of 2,978 census tracts were examined.

### Variables

Standardized mortality rates (SMR), stratified by sex, were studied for four mortality causes: tumor of the bronquial, lung and trachea (ICD-10: C33-C34), diabetes mellitus (ICD-10:E10-E14), breast cancer (ICD-10:C50), and prostate cancer (ICD-10:C61). The mortality data was provided from the Catalan Mortality Registry. We only used death certificates of residents of the Metropolitan Region of Barcelona who died between 1994 and 2007.

### Deprivation index

Socioeconomic conditions were summarized using a DI in a census tract level. Sixteen socio-demographic variables available in the Spanish CPH were included. Four of these: unemployment rate, percent with low educational level, manual workers and temporary workers, have been used in several DIs for small-area studies in Spain [46,50]. The other variables (university education, mono-parental homes, activity rate, immigrants, homes without heating, no toilet/bathroom in the home, bad communications, vandalism/crime, few green areas, exterior noise, contamination/bad smells, and dirty street) were chosen based on literature that focused on the association between contextual indicators and their effect on health, particularly those that analyzed gender differences [38]. We chose contextual indicators, most of them subjective, which were representative of the neighborhood characteristics (Table 1).

**Table 1:** Mean and Percentile distribution of socioeconomic indicators over census tracts of the Barcelona Metropolitan Region, 2001

	**Women**				**Men**			
**Indicators (%)**	**P**_**25**_	**P**_**50**_	**P**_**75**_	**Mean**	**P**_**25**_	**P**_**50**_	**P**_**75**_	**Mean**
**Manual workers**	36.8	53.3	67.2	51.6	41.0	58.7	73.2	56.0
**Unemployment**	5.2	6.2	7.4	6.4	4.8	6.0	7.4	6.3
**Temporary workers**	16.7	20.1	24.4	20.9	13.8	17.2	21.2	18.0
**Insufficient education**	10.3	15.8	22.8	17.1	6.4	10.8	16.3	12.0
**University education**	26.4	32.2	37.9	32.1	25.0	31.9	39.1	32.4
**Monoparental homes**	10.3	20.0	32.4	23.1	0.0	16.7	33.3	22.0
**Activity rate**	53.4	56.8	60.6	57.1	63.1	67.1	71.3	67.3
**Inmigrants**	1.8	3.1	5.3	4.1	1.7	3.3	6.0	4.7
**Homes without heating**	64.8	79.7	90.0	75.3	64.8	79.7	90.0	75.3
**No toilet/bathroom in home**	0.6	1.0	1.7	1.4	0.6	1.0	1.7	1.4
**Bad communications**	3.3	6.1	14.3	11.5	3.3	6.1	14.3	11.5
**Vandalism/Crime**	19.0	26.3	38.7	31.3	19.0	26.3	38.7	31.3
**Few green areas**	22.0	35.0	50.6	37.0	22.0	35.0	50.6	37.0
**Exterior noise**	32.9	40.4	48.0	40.6	32.9	40.4	48.0	40.6
**Contamination/Bad smells**	17.0	23.6	31.5	25.2	17.0	23.6	31.5	25.2
**Dirty streets**	30.8	38.2	47.4	39.7	30.8	38.2	47.4	39.7

P_**25**_: 25 Percentile; P_**50**_: 50 Percentile; P_**75**_: 75 Percentile; Min: Minimum; Max: Maximum.

The DI was constructed by aggregating the above-mentioned variables using another indicator, DP_2_, instead of the standard PCA method. DP_2_ is an iterative procedure that weights partial indicators depending on their correlation with the global index [41,45,51]. It is able to capture all the non-redundant variance of the indicators (i.e. avoiding multicollinearity); thus, allowing for the inclusion of a greater number of variables [41,42]. Given that DP_2_ uses all the valuable information contained in the partial indicators the more complete the final DI will be, since each variable contains unique information not present in others [44]. Magnifying the goodness of DP_2_ versus the extraction of an index through principal component analysis, especially when introducing a greater number of variables would not suffice for identifying a single component for the index. More than one component should be collected when applying PCA thus resulting in an arbitrary choice of weights for aggregating the components to obtain the index. Pena [41] and Zarzosa [42] showed that DP_2_ fulfills all the properties of a good composite indicator; monotony, unicity, invariance, homogeneity, transitivity, exhaustivity, existence and determination, and additivity.

The process for aggregating the variables using DP_2_ is a four-step process previously described in detail [51].

### Data analysis

When spatial data is available, the variability in the observed response is usually greater than the expected, resulting in over dispersion. In fact, it is important to distinguish between two sources of extra-variability [52]. The first most important source is the so-called 'spatial dependence’; it is a consequence of the correlation between the spatial unit and the neighboring spatial units, generally contiguous geographic areas. Nearby areas are more similar than those far apart. Part of this dependence is not really a structural dependence, but mainly due to variables not included in the analysis. The second source is the independent extra variability, spatially uncorrelated, called heterogeneity (not spatial); it is the result of unobserved variables, without spatial structure, which could influence the response [52,53].

To account for this extra variability, it is necessary to introduce some structure in the model. Otherwise, unless the model is linear, the estimates of the parameters of interest and the standard errors of the estimators will be biased; therefore any inference based on them, will be wrong [54].

In this paper we have chosen to use hierarchical Bayesian models, more specifically a model based on Besag, York and Mollié [55,56] (BYM) to analyze the relationship between mortality and small-area deprivation.

As we know, the idea is to introduce two random effects in a generalized linear model with Poisson response, in order to capture the extra-variability [53].

(1) $$\begin{array}{l} O_{i}∼Poisson\left(\mu_{i}Pop_{i}\right)\\ Log\left(\mu_{i}\right)=\alpha +Log\left(Pop_{i}\right)+\sum_{k=1}^{4}\beta_{k}IndexQ_{\mathrm{ki}}+\upsilon_{i}+S_{i}+\beta_{5}Pop4564_{i}+\beta_{6}Pop65m_{i} \end{array}$$

Where O_i_ denotes the observed cases of the response variable in the census tract i, *μ*_*i*_ is the relative risk in section i, Pop_i_ is the population (men or women) of the census tract, *υ*_*i*_ is the random effect which reflects the heterogeneity; S_i_ is the random effect that reflects the spatial dependence, α is the intercept, interpreted as the logarithm of the baseline risk; IndexQ_ki_ is the quintile of the deprivation index (standardized) in the census tract i (the first quintile was taken as a reference); the β are the parameters of the model, which can be interpreted as the logarithms of the relative risks associated with the explanatory variables; Pop4564_i_ is the percentage of men (or women) from 45 to 64 years in the census tract i, and Pob65m_i_ is the percentage of men (or women) aged 65 years or older in the census tract i.

The DI was categorized into quintiles because the influence of deprivation on the (spatial) variation of mortality could be non-linear.

The non-spatial random effect, also called heterogeneity, assumed to be normally distributed, with a mean of zero and constant variance. For the spatial random effect a conditional autoregressive model (CAR) is used [57,58]. This approach, which is the most utilized and has the lowest computational cost, approximates the spatial dependence as an average spatial effect of neighboring areas [53]. The areas considered are the census tracts of each municipality in the Metropolitan Region of Barcelona and surrounding areas are defined as adjacent census tracts, in other words they share a border.

Note that the specified model does not use, as an offset the number of cases expected in the census tract, but the population (men or women) of the same. This is because, unlike the standard BYM model [53], here we use the crude death rate (from the census tract) and not the standardized mortality ratio as an indicator of mortality. The reason is to avoid the problem called 'mutual standardization' [59,60]. Rosenbaun and Rubin [59] show how the use of standardized rates as the response variable in ecological regression models leads to biased results if only the answer, not predictors, are adjusted for the same confounder, usually the age distribution. When the predictor is not adjusted, it is implicitly assumed that the effect of predictor is constant for all strata of the confounding variable. This may be true for the effect of an air pollutant, in principle it is the same for all ages, but not for the deprivation index. Grisotto *et al.*[60], in line with Rosenbaun and Rubin [59] show that unbiased estimators can be obtained by adjusting the response and the predictors (the index of deprivation in this case) with the same variable (age distribution) or, even easier to use are crude rates as the response variable and entering age (as an average or structured) as an explanatory variable of the model. This is why we have introduced the age structure of the census tract (proportion of men and women aged 45 to 64 years and 65 years or more). The introduction of age also lets you control the age effect in the model. This modification allows to overcome methodological limitations of the standard implementation of the BYM model [18]. Furthermoe, and also getting no significance for the coefficients of the variables that represent the population from 45 to 64 years and older population or more at 65, none of the cases studied, we can say that the risks found not differ by age group. Moreover, because the estimates associated with variables representing the population aged 45 to 64 years and older population less than 65, were not significant in any of the cases studied, we can say that risks not found differ by age group.

A second difference from the standard BYM model, not so obvious, is the use of standardized explanatory variables. The reason is that the spatial random effect, approximated by CAR, can be correlated with some (or all) explanatory variables that have a similar spatial dependence (known as concurvity). If this were the case, there would be an over-adjustment, unlike the phenomenon of multicollinearity in linear models, which could bias the estimates [61]. Simulations performed by us suggest that the problem could be solved by completely standardizing the potentially problematic explanatory variable. On the other hand, the introduction of the deprivation index (standardized) into quintiles, in addition to collecting a possible nonlinear effect, could mitigate much of the problem.

Spatial models were built as Bayesian hierarchical models with two stages [62] and estimated using the integrated nested Laplace approximation [62-64] (see Appendix: Annex).

Models were compared using the DIC (Deviance Information Criterion) [65] and the conditional predictive ordinate (CPO) for each observation (in fact –*mean*(log(*cpo*)) [66,67]. CPO is a cross-validated predictive approach i.e., predictive distributions conditioned on the observed data with a single data point deleted. Asymptotically the CPO statistic has a similar dimensional penalty as DIC. In this perspective, the CPO statistic may be similar to DIC. In both cases, the lower the DIC or the CPO, the better the model.

There was not experimental research in this work. All computations were carried out using the interface INLA [68], running directly in R (version R 2.11.0) [69].

## Results and discussion

According to the Spanish CPH of 2001 the average number of inhabitants per census tract in the metropolitan region of Barcelona was 1201.33 (standard deviation 526.55; median 1085.50).

Table 1 describes de distribution of the socioeconomic indicators, used to construct the DI, by gender in the Metropolitan Region of Barcelona in 2001. We observed a high percent of temporary workers (20.9% women, 18.0% men) and manual workers (51.6% women, 56.0% men) for both men and women. Women had a higher rate of insufficient education (17.1% vs. 12%) and a lower activity rate (57.1% vs. 67.3%) than men. Over a third of the individuals claimed their neighborhoods had dirty streets, exterior noise, few green areas and vandalism/crime.

Tables 2 and 3 show the associations between cause-specific mortality and deprivation by quintiles of the index, for men and women controlled for age. In the case of women (Table 2), we observed a positive association for diabetes mortality. However, both lung cancer mortality (RR in Q5 = 0.78; 95%CI: 0.65-0.93) and breast cancer mortality (RR in Q5 = 0.81; 95%CI: 0.71-0.92) had an inverse relationship with socioeconomic deprivation; women with greater deprivation had less mortality risk (statistical significance was present only in the fifth quintile).

**Table 2:** Association between three mortality causes and the socioeconomic deprivation index in women

	**Trachea, bronchial and lung cancer**	**Diabetes**	**Breast cancer**
**Deprivation (Quintiles)**	**RR (95% CI)**^1^	**RR (95% CI)**^1^	**RR (95% CI)**^1^
**log RR Q**_**2**_	0.87 (0.75 - 1.01)	1.27 (1.13 - 1.42)	0.99 (0.89 - 1.11)
**log RR Q**_**3**_	0.86 (0.73 - 1.00)	1.25 (1.11 - 1.41)	1.00 (0.89 - 1.12)
**log RR Q**_**4**_	0.92 (0.78 - 1.08)	1.22 (1.07 - 1.38)	0.93 (0.82 - 1.04)
**log RR Q**_**5**_	0.78 (0.65 - 0.93)	1.24 (1.08 - 1.42)	0.81 (0.71 - 0.92)
**Random Effects (σ)**	**Mean (SD)**^2^	**Mean (SD)**^2^	**Mean (SD)**^2^
**Spatial (**${\hat{\sigma }}_{S}$**)**	0.35 (0.07)	0.34 (0.06)	0.31 (0.06)
**Heterogeneity (**${\hat{\sigma }}_{\upsilon }$**)**	0.58 (0.03)	0.56 (0.02)	0.54 (0.02)
**Mean (log(CPO))**	1.177083	1.714764	1.736699
**Deviance Information Criterion (DIC)**	6875.091	10070.06	10270.72
**Effective Number or Parameters**	602.7834	1079.27	1054.834

^1^ Mean (95% Credible Interval).

^2^ Mean (Standard Deviation).

Credible interval does not contain the unity.

RR Relative Risk.

**Table 3:** Association between three mortality causes and the socioeconomic deprivation index in men

	**Trachea, bronchial and lung cancer**	**Diabetes**	**Prostate cancer**
**Deprivation (Quintiles)**	**RR (95% CI)**^1^	**RR (95% CI)**^1^	**RR (95% CI)**^1^
**log RR Q**_**2**_	1.19 (1.10 -1.29)	1.17 (1.03 - 1.33)	1.13 (1.00 - 1.26)
**log RR Q**_**3**_	1.23 (1.13 - 1.34)	1.17 (1.03 - 1.33)	1.09 (0.96 - 1.23)
**log RR Q**_**4**_	1.22 (1.12 -1.33)	1.08 (0.94 - 1.24)	0.96 (0.85 - 1.09)
**log RR Q**_**5**_	1.31 (1.19 -1.43)	1.09 (0.94 - 1.26)	0.91 (0.79 - 1.04)
**Random Effects (σ)**	**Mean (SD)**^2^	**Mean (SD)**^2^	**Mean (SD)**^2^
**Spatial (**${\hat{\sigma }}_{S}$**)**	0.26 (0.04)	0.31 (0.06)	0.38 (0.06)
**Heterogeneity (**${\hat{\sigma }}_{\upsilon }$**)**	0.49 (0.01)	0.57 (0.02)	0.54 (0.02)
**Mean (log(CPO))**	2.48789	1.507189	1.674478
**Deviance Information Criterion (DIC)**	14209.69	8818.788	9843.01
**Effective Number or Parameters**	1614.22	872.0202	1001.67

^1^ Mean (95% Credible Interval).

^2^ Mean (Standard Deviation).

Credible interval does not contain the unity.

RR Relative Risk.

For men (Table 3), we observed a positive association for lung cancer mortality; where, at greater socioeconomic deprivation there was greater mortality risk (RR in Q5 = 1.31; 95%CI: 1.19-1.43). For diabetes mortality we also observed a positive association with mortality risk and deprivation; however, statistical significance was found only in second and third quintiles. On the other hand, for prostate cancer mortality there is no systematic relationship between deprivation and mortality risk. Only the second quintile is significant with a RR = 1.13 (95% CI: 1.00-1.26).

Overall, our results are consistent with those provided by the literature. At greater deprivation, there is an increased risk of dying from diabetes for both sexes (although in the case of men the relative risks associated with the third quintile of deprivation onwards were not statistically significant) and of dying from lung cancer for men. On the other hand, at greater deprivation, there is a decreased risk of dying from breast cancer and lung cancer for women (although in both cases only the relative risk associated with the top quintile of deprivation was statistically significant). We did not find a clear relationship in the case of prostate cancer (presenting an increased risk but only in the second quintile of deprivation).

This study has been able to improve the statistical methodology applied in building deprivation indices as well as the robustness of spatial adjustments in ecological studies. Our results were consistent with the existing literature that analyzes the association between deprivation and mortality (lung cancer, diabetes mellitus, breast cancer and prostate cancer); however, our focus was in urban small-areas. As some literature suggests, mortality differs in rural versus urban settings, particularly in women [70]; thus, it would be important to explore geographic differences in more detail in future studies. In addition, future studies should also focus on researching these same causes of death in other European cities and additional causes of death in the Barcelona Metropolitan Region.

## Conclusions

We believe our results were obtained using a more robust methodology. First off, we have built a better index that allows us to directly collect the variability of contextual variables without having to use arbitrary weights, as is assumed by the aggregation of components through PCA extraction. Building an index using PCA is adequate, the results do not differ from those obtained by DP_2_, when you add only a few variables because the first component allows you to collect enough variability. When using a large number of variables, like shown in this article, it is clear that a single component is not enough to collect the variability contained in the variables and the index should thus be constructed by aggregating two or more components. The arbitrariness in the choice of aggregation weights is what we avoided by using the DP_2_ methodology as the algorithm used for constructing the index naturally allows you to collect all the variability contained in the variables excluding only the shared part and not the variable itself.

Secondly, we have solved two major problems that are present in spatial ecological regressions, i.e. those that use spatial data and, consequently, perform a spatial adjustment in order to obtain consistent estimators. In particular, the problem of mutual standardization and the problem of concurvity. As stated previously, the problem of concurvity is present when the spatial random effect is correlated with some (or all) of the explanatory variables that have similar spatial dependence. Using crude rates as indicators of mortality for the response variable and the introduction of age as an explanatory variable in the model allows us to ensure the collection of unbiased estimators. Furthermore, the introduction of age in the model allows us to monitor its effect on the results. On the other hand, the introduction of the index (standardized) into quintiles, in addition to collecting a possible nonlinear effect, could mitigate much of the problem of concurvity.

One limitation of this study is the limited variable selection due to lack of statistical information in censuses. Additionally, the deprivation index was computed using information from the 2001 Census of Population and Housing (the only information available) while mortality data was recorded using information from the period 1994–2007. Some characteristics recorded in 2001 may not capture the real environment of the areas in the pass, when the deaths occurred. However, the data collection period for the construction of the index corresponds to the half point of the period in which the mortality data was collected and the relative position of the census track with respect to the deprivation index (i.e. the quintile of the index where it was located) remains very stable in time. It is also important to point out that given that the data is over a decade old the present patterns of associations may have changed, primarily due to the increased amount of immigration since 2001 and the activity rate downfall due to the economic crisis.

## Appendix

### Annex

Spatial models were built as Bayesian hierarchical models with two stages [62,63]. The first stage was the observational model $p\left(y|x\right)$, where *y* denoted the vector of observations and *x* are the unknown parameters following a Gaussian Markov random field (GMRF) denoted as $p\left(x|\theta \right)$. The second stage was given by the hyperparameters *θ* and their respective prior distribution $p\left(\theta \right)$. The desired posterior marginals

(2) $$p\left(x_{i}|y\right)=\int_{\theta }p\left(x_{i}|\theta ,y\right)p\left(\theta |y\right)d\theta$$

of the GMRF were approximated using the finite sum

(3) $$\tilde{p}\left(x_{i}|y\right)=\sum_{k}\tilde{p}\left(x_{i}|\theta_{k},y\right)\tilde{p}\left(\theta_{k}|y\right)\Delta_{k}$$

where $\tilde{p}\left(x_{i}|\theta ,y\right)$and $\tilde{p}\left(\theta |y\right)$ denoted approximations of $p\left(x_{i}|\theta ,y\right)$ and $p\left(\theta |y\right)$, respectively. The finite sum (A1) was evaluated at support points $\theta_{k}$ using appropriate weights $\Delta_{k}$.

The posterior marginal $p\left(\theta |y\right)$ of the hyperparameters is approximated using a Laplace approximation [71].

(4) $$\tilde{p}\left(\theta |y\right)\propto \frac{p\left(x,\theta ,y\right)}{{\tilde{p}}_{G}\left(x|\theta ,y\right)}|x=x^{*}\left(\theta \right)$$

where the denominator ${\tilde{p}}_{G}\left(x|\theta ,y\right)$ denoted the Gaussian approximation of $p\left(x|\theta ,y\right)$ and $x^{*}\left(\theta \right)$ was the mode of the full conditional $p\left(x|\theta ,y\right)$[72].

According to Rue *et al.*[63], it is sufficient to “numerically explore" this approximate posterior density using suitable support points $\theta_{k}$ in (A1). In this paper, these points were defined in the h-dimensional space, using the strategy called central composite design. Here, centre points were augmented with a group of star points, which allowed for estimating the curvature of $\tilde{p}\left(\theta |y\right)$.

Here, to approximate the first component of (A1) a simplified Laplace approximation (less expensive from a computational point of view with only a slight loss of accuracy) was used [62-64].

## Abbreviations

DI, Deprivation index; PCA, Principal component analysis; DP2, Pena distance; OR, Odds ratio; DM2, Diabetis mellitus type 2; BMI, Body mass index; RR, Relative risk; 95% CI, 95% confidence interval; CPH, Census population and housing; SD, Standard deviation; SMR, Standardized mortality rates; ICD-10, International classification of disease v. 10; BYM, Besag, York and Mollié model; CAR, Conditional autoregressive model; DIC, Deviance information criterion; CPO, Conditional predictive ordinate.

## Competing interests

There are no conflicts of interest for any of the authors. All authors disclose any financial and personal relationships with other people or organizations that could inappropriately influence and/or bias their work.

## Authors’ contributions

All authors contributed equally to the development of the manuscript. MS had the idea of writing this manuscript, based on the Masters thesis of NS. NS and CS were responsible for choosing the variables and collect data. CS and MS specified the models. MS and BB were responsible for the statistical analyzes. Interpretation of the results were done mainly by CS, NS and MS. Drafting of the manuscript were done by CS, MS and NS. All authors revised and approved the final version of the manuscript.

## Pre-publication history

The pre-publication history for this paper can be accessed here:

http://www.biomedcentral.com/1471-2458/12/574/prepub
